# Sensitive detection of hydrocarbon gases using electrochemically Pd-modified ZnO chemiresistors

**DOI:** 10.3762/bjnano.8.9

**Published:** 2017-01-10

**Authors:** Elena Dilonardo, Michele Penza, Marco Alvisi, Gennaro Cassano, Cinzia Di Franco, Francesco Palmisano, Luisa Torsi, Nicola Cioffi

**Affiliations:** 1 Department of Chemistry, Università degli Studi di Bari Aldo Moro, Bari, Italy; 2 Department of Electrotechnics and Electronics, Politecnico di Bari, Bari, Italy; 3 Italian National Agency for New Technologies, Energy and Sustainable Economic Development (ENEA), Laboratory Functional Materials and Technologies for Sustainable Applications - Brindisi Research Center, Brindisi, Italy; 4 CNR-IFN Bari, Bari, Italy

**Keywords:** chemiresistive gas sensor, electrosynthesis, hydrocarbon gas sensor, Pd-modified ZnO, ZnO nanorods

## Abstract

Pristine and electrochemically Pd-modified ZnO nanorods (ZnO NRs) were proposed as active sensing layers in chemiresistive gas sensors for hydrocarbon (HC) gas detection (e.g., CH_4_, C_3_H_8,_ C_4_H_10_). The presence of Pd nanoparticles (NPs) on the surface of ZnO NRs, obtained after the thermal treatment at 550 °C, was revealed by morphological and surface chemical analyses, using scanning electron microscopy and X-ray photoelectron spectroscopy, respectively. The effect of the Pd catalyst on the performance of the ZnO-based gas sensor was evaluated by comparing the sensing results with those of pristine ZnO NRs, at an operating temperature of 300 °C and for various HC gas concentrations in the range of 30–1000 ppm. The Pd-modified ZnO NRs showed a higher selectivity and sensitivity compared to pristine ZnO NRs. The mean sensitivity of Pd-modified ZnO NRs towards the analyzed HCs gases increased with the length of the hydrocarbon chain of the target gas molecule. Finally, the evaluation of the selectivity revealed that the presence or the absence of metal nanoparticles on ZnO NRs improves the selectivity in the detection of specific HCs gaseous molecules.

## Introduction

Hydrocarbons (HCs) are molecules consisting of carbon and hydrogen atoms, and the gaseous species can be present in the atmosphere depending on their volatility or vapor pressure. As volatile molecules in the atmosphere, they are classified as volatile organic compounds (VOCs). The U.S. Environmental Protection Agency (EPA) defines a VOC as any carbonaceous compound of carbon that is involved in atmospheric photochemical reactions [1].

The presence of HCs in the atmosphere has either anthropogenic or natural sources. The former involve the emission of a great number of species and concern various industrial activities, mostly related to production, treatment, storage and combustion of fossil fuel. The natural sources produce a much smaller variety of HC species, indeed the major emitted HC gas is methane [1]. Therefore, it is clear that the detection and monitoring of gaseous HCs is fundamental for environmental protection [2–3]. The direct exposure to HCs can negatively affect the human health, from irritation of the respiratory system to cancer [4–5]. Therefore, the selective detection of particular gaseous HCs in a complex matrix is one of the challenges in gas detection for environmental monitoring.

In the last years, various techniques have been used for HC gas detection [6–9]. However, they still have some limitations such as the need for expensive instruments, time-consuming procedures, complicated pre-treatments and periodical maintenance [10–14]. Since a precise monitoring of HCs even at low concentrations can be beneficial to preserve the environment and human health, the improvement of cost-effective HCs gas sensors, including networked sensor-systems and new strategies for hydrocarbon sensing, is a matter of interest for the scientific community.

HCs gas sensors based on organic conducting polymers (such as polyaniline (PANI) [15–16], polypyrrole (PPy) [17] and polythiophene (PTh) [18]) and on carbon-based nanomaterials with desired functionality and conductivity (e.g., carbon nanotubes (CNTs) [19] and graphene [20]) exhibit a comparably good gas-sensing performance [21–22]. However, due to their high affinity toward HCs and low thermal stability, they are sometimes unstable and exhibit poor sensitivity [23–24]. In this context, metal oxides (MO*_x_*) have been proposed as promising active sensing layers because of their advantageous properties such as good sensitivity under ambient conditions and easy preparation [25].

The fundamental process of the gas-sensing mechanism, holding the MO*_x_*-based sensing material at elevated temperatures above 300 °C, is the reaction of the surrounding gases with the oxygen of the MO*_x_* layer, causing changes in the surface potential and resistivity of the sensing material. The electrical resistance can increase or decrease, depending on the type of doping of MO*_x_* (p- or n-type) and on the analyte gas. There are oxidizing gases, such as nitrogen oxide (NO_2_), and ozone (O_3_), and reducing gases such as carbon monoxide (CO) and hydrocarbons (HCs) [26]. The magnitude of the variation of the electrical resistance gives a direct measure of the concentration of the analyte gas [25].

In the last decades, different nanostructured MO*_x_*-based gas sensors with improved performance in the HC gas detection were developed [27–37]. Among MO*_x_* semiconductors, nanostructured ZnO is promising as sensing material in chemiresistive gas sensors, although its use still reveals some drawbacks related to its low selectivity, long response and recovery times, high power consumption, and poor stability over the time [38]. These limits can be overcome by functionalization of ZnO nanostructures with noble metal nanoparticles. Specifically, Pt and Pd are widely applied for monitoring explosive and toxic gases. The catalytic metals do not change the free energy of the reactions but lower the activation energy. The sensing response of ZnO towards most of the toxic gases in general, and towards HC gases in particular, can be improved by surface deposition of noble metals. Sivapunniyam et al. [39] have reported the improvement of ZnO-nanorod-based HC gas sensing by doping the metal oxide with Pt nanoparticles. Gurav et al. [40] reported an improvement of 60% in the response towards LPG detection at an operating temperature of 498 K using ZnO nanorods functionalized by catalytic Pd NPs. Moreover, the Pd-sensitized vertically aligned ZnO nanorods showed higher selectivity towards LPG than to CO_2_.

Most of the processes that, to date, have been developed to functionalize MO*_x_* nanostructures with noble metal NPs [32,41] are unique and effective, but, at the same time, also complex and time-consuming. Moreover, metal NPs deposited by these processes can undergo undesired clustering with the subsequent worsening of their catalytic activity [10]. Therefore, various new synthetic procedures have been proposed to overcome these limits [42–43].

In this study, we propose a one-step strategy based on sacrificial anode electrolysis (SAE) to synthesize stabilized Pd NPs [44], directly deposited on the surface of sol–gel pre-synthesized ZnO nanostructures. Further post-annealing at 550 °C to obtain ZnO NRs is necessary [45].

The prepared hybrid Pd@ZnO nanostructures are proposed as active layer in chemiresistive gas sensors for the detection of pollutant HCs. The effect of the Pd catalyst on the performance of ZnO-based gas sensors was investigated by the comparison of the gas sensing results of pristine and Pd-modified ZnO NRs, at an operating temperature of 300 °C, towards methane (CH_4_), propane (C_3_H_8_) and butane (C_4_H_10_) at a wide range of gas concentrations (30–1000 ppm). Pd-modified ZnO NRs showed a higher selectivity and sensitivity compared to pristine ZnO NRs. Moreover, the mean sensitivity of Pd@ZnO NRs towards the analyzed HCs gases increased with the length of the hydrocarbon chain of the target gas molecule.

Finally, the gas-sensing measurements towards interfering gaseous pollutants (e.g., NO_2_) revealed that the presence of Pd NPs on the surface of ZnO improves the selectivity in the detection of specific gaseous molecules. Specifically Pd@ZnO chemiresistors showed a high selectivity towards HCs compared to the pristine ZnO-based gas sensors. On the contrary, high selectivity towards NO_2_ gas detection was obtained by using pristine ZnO chemiresistors.

## Experimental

### Sol–gel synthesis of ZnO

ZnO nanostructures were prepared via a sol–gel process following the procedure reported in [45]. The subsequent washing of the obtained gel led to the complete removal of chlorine ions in the liquid phase. Finally, a thermal treatment at 120 °C for 2 h allowed us to maintain hydroxyl (–OH) groups on the oxide surface in order to permit the attachment of Pd NPs during the electrochemical deposition process [45].

### Electrochemical decoration of ZnO by Pd NPs

Pd@ZnO nanostructures were prepared by SAE as reported in [44], but in this case Pd foils were used as anode (working electrode) to obtain colloidal Pd NPs. Tetraoctylammonium bromide (TOAB) was simultaneously used as electrolyte and stabilizer for Pd NPs, at a concentration of 0.05 M in 5 mL in a solution of tetrahydrofurane (THF)/acetonitrile (ACN) (3:1 ratio).

Electrolysis was performed following the experimental conditions reported in [46]. Further, Pd@ZnO nanostructures were centrifuged (6000 rpm) to separate the unsupported colloidal Pd NPs from the heavier Pd@ZnO hybrid systems. Subsequently, unfunctionalized ZnO and Pd@ZnO hybrids were annealed at 550 °C for 2 h in air to obtain pristine and Pd-modified rod-like ZnO NRs.

### Material characterization

The chemical characterization of the surface of pristine and functionalized ZnO NRs was performed by a Thermo VG Theta Probe XPS spectrometer, using a micro-spot monochromatic Al Kα source in a fixed analyzer transmission mode. The survey spectrum was acquired with 150 eV pass energy, and high-resolution spectra with 100 eV pass energy. The reproducibility was evaluated replicating the analysis in five different points on each sample.

TEM (FEI TECNAI T12 TEM instrument operated at 120 KV) and SEM (field emission Zeiss ΣIGMA SEM operated at 5–10 KV, 10 μm aperture) analyses were performed to evaluate the morphology of pristine and Pd-modified ZnO composites.

### Preparation of chemiresistive sensors and gas-sensing set-up

**Figure 1:**
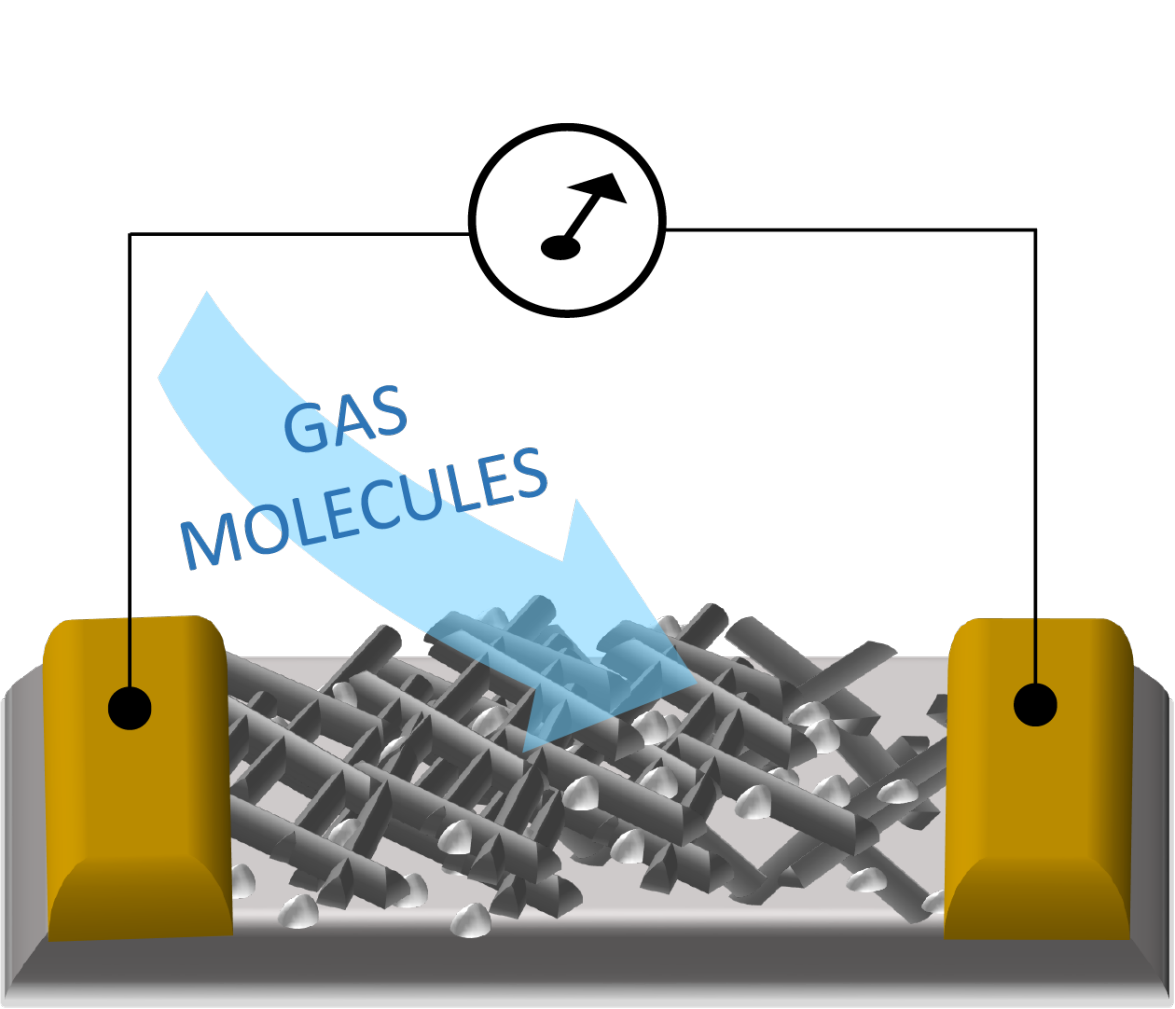
A scheme of a Pd-modified rod-like ZnO-based chemiresistive gas sensor.

Figure 1 shows the scheme of the used Pd-modified rod-like ZnO-based chemiresistive gas sensor.

After the annealing at 550 °C, pristine and Pd-modified ZnO were redispersed in ACN and drop-cast on alumina substrates to obtain sensing layers between gold contacts. These assemblies were subsequently thermally stabilized at 300 °C for 2 h. The description of the used experimental set-up for gas sensing analyses is reported elsewhere [47]. The reference and the carrier gas, used to dilute the gaseous analyte keeping the total flow constant at 1000 sccm, was dry air. The gas sensing measurements were performed by evaluating the resistance variation of the active layer during the exposure to the analyte gas at a sensor temperature of 300 °C. Each sensing cycle consisted of an initial step of 60 min to stabilize the sensor signal under the reference gas, the exposure to decreasing concentrations of the target gas for 10 min. (For the gas response measured after 2 months to evaluate the sensor stability over time and a possible effect of exposure time, the exposure time was 20 min.) The exposure steps were separated by 30 min of recovery to restore the signal to the initial value and to clean the sensor surface under the reference gas flow. The response and recovery times were defined as the time needed to reach 90% of the resistance saturation value under the exposure to the analyzed gas and the time needed to recover 10% of the original resistance value in air after the exposure to the gas, respectively.

The sensor response is reported as Δ*R*/*R*_i_ (%), and the mean gas sensitivity, *S*_m_ (%·ppm^−1^), is defined as the weighted mean of relative change of resistance (%) divided by gas concentration unit (ppm) [45].

## Results and Discussion

### Chemical and structural properties

**Figure 2:**
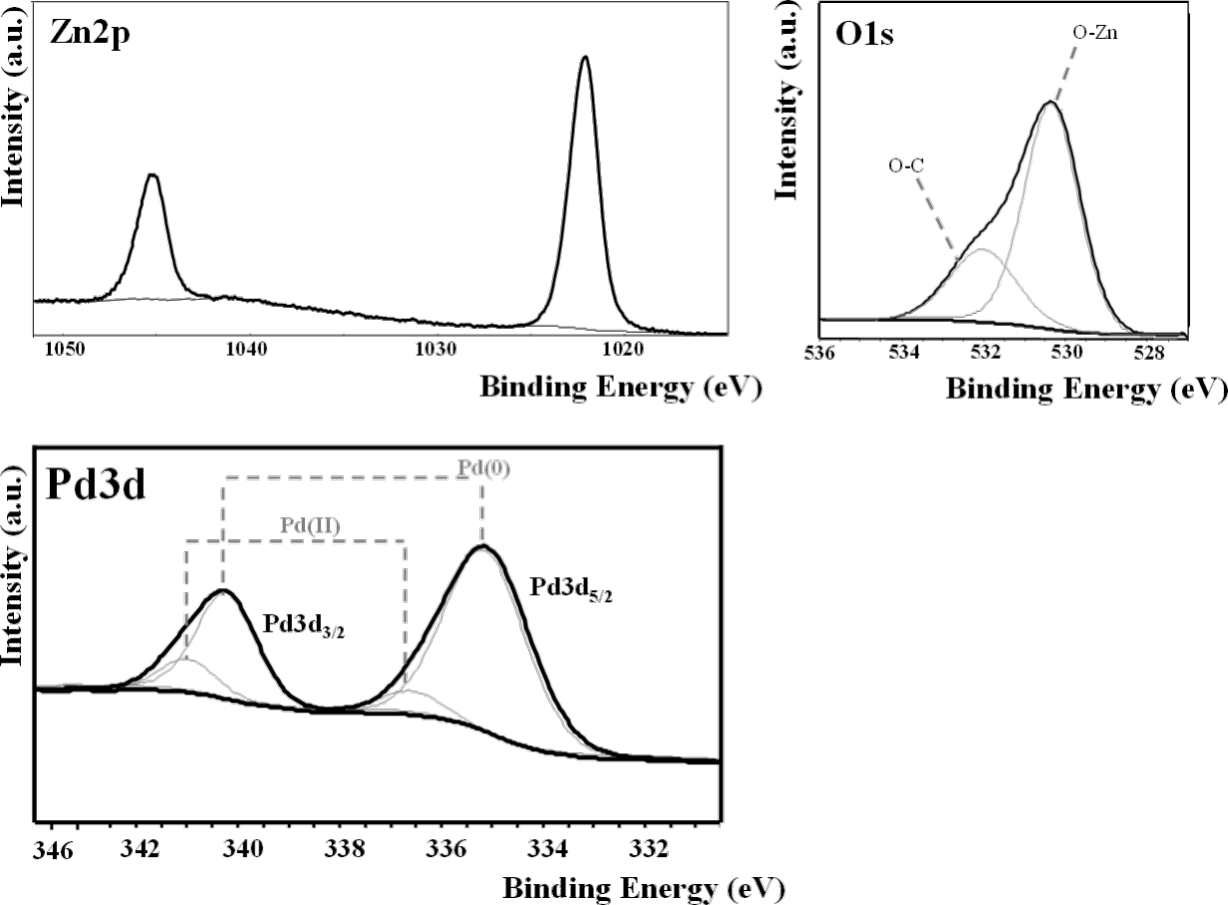
XPS spectra of the chemical elements in pristine ZnO: Zn 2p and O 1s spectra, deconvoluted in two components (O–Zn and O–C), with the additional Pd 3d spectrum in Pd@ZnO.

The chemical composition of the surface of pristine and Pd-modified ZnO NRs was evaluated by XPS analysis. In Figure 2 the high-resolution XPS spectra of Zn 2p and O 1s in pristine ZnO, and of Pd 3d in Pd@ZnO hybrid structures are reported.

High-resolution XPS Zn 2p and O 1s spectra are the same in pristine and functionalized ZnO. Moreover, in both cases, the relative area of the two components of the O 1s spectrum, O–Zn and O–C, remains unchanged after the surface functionalization. Therefore, the atomic ratio O–Zn/Zn (the percentage of oxygen bound to metal divided by the total metal percentage) remained stoichiometric, i.e. equal to 1, also after the metal decoration, as reported in Table 1.

**Table 1:** XPS surface chemical composition of pristine and Pd-functionalized ZnO NRs, annealed at 550 °C. The value for O–Zn refers to the atomic percentage of oxygen bound to zinc.

	ZnO	Pd@ZnO

C	15.7% ± 0.5%	13.5% ± 0.5%
O_(total)_	44.5% ± 0.5%	44.5% ± 0.5%
O–Zn	39.6% ± 0.5%	40.9% ± 0.5%
Zn	39.8% ± 0.5%	41.0% ± 0.5%
Pd	—	1.0% ± 0.2%

In Pd@ZnO, the presence of palladium confirmed the successful electrochemical decoration of ZnO nanostructures. The Pd 3d high-resolution XPS spectrum is reported in Figure 2. The signal is composed of two doublets. The first one, Pd 3d_5/2_, at 335.3 ± 0.1eV, was attributed to nanostructured elemental palladium [46]. The second doublet, Pd 3d_5/2_ at 337.0 ± 0.1eV, was attributed to Pd(II) species, probably due to the presence of low amounts of PdO at 336.5 ± 0.1 eV [46].

Table 1 reports the surface atomic percentages of pristine and Pd-functionalized ZnO NRs after annealing at 550 °C. The total amount of palladium deposited on ZnO was about 1.0 atom %. The TOAB surfactant was almost completely removed from the Pd surface after annealing.

**Figure 3:**
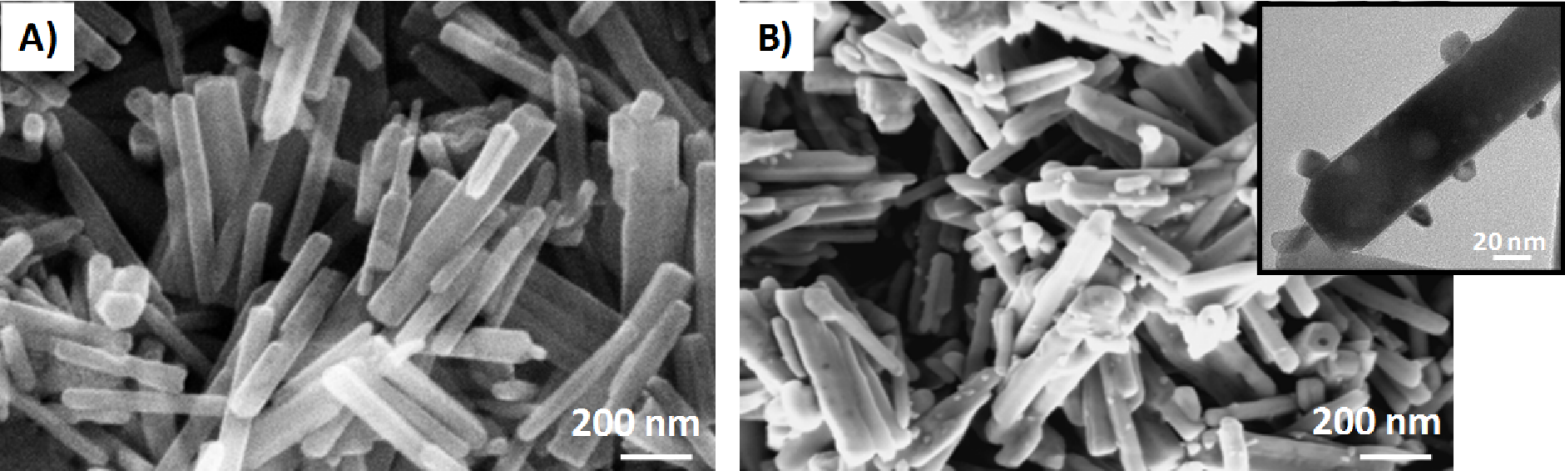
SEM images of **A)** pristine and **B)** Pd-modified ZnO nanostructures, after thermal annealing at 550 °C. The inset shows the TEM image of Pd@ZnO NRs.

In Figure 3, the SEM images of pristine and Pd-functionalized ZnO NRs after thermal annealing at 550 °C are reported. Pristine and functionalized ZnO reveal a rod-like shape with an average diameter of about 30 nm and length of about 500 nm. In the case of modified ZnO, single Pd NPs of about 15 nm in diameter are evident on the NR surface, as reported also in the TEM inset of Figure 3B, confirming the successful electrochemical functionalization of ZnO NRs.

The presence of Pd NPs on ZnO NRs strongly affects gas adsorption and reactivity and, hence, the gas sensing as discussed in the following section.

### Gas-sensing performance

**Figure 4:**
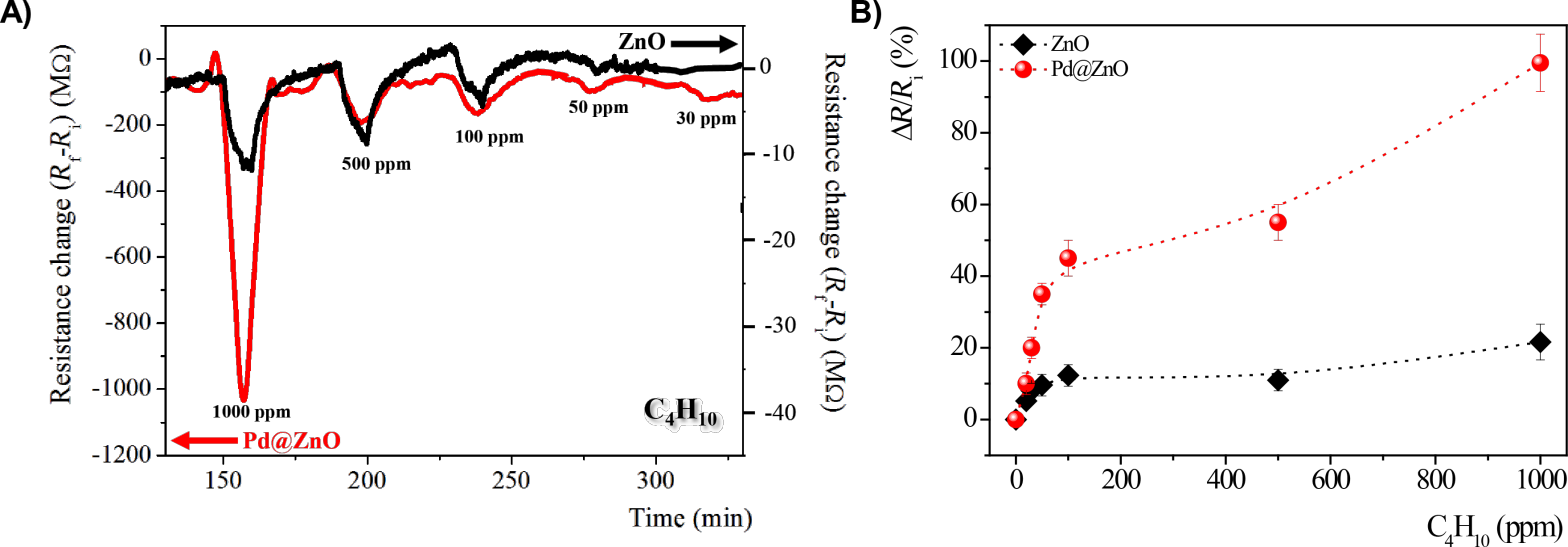
A) Time response and B) calibration curves of the change of electrical resistance of chemiresistors based on pristine and Pd-modified ZnO NRs, exposed to different concentrations (30–1000 ppm) of butane (C_4_H_10_) at an operating temperature of 300 °C.

Figure 4A shows the time responses of the electrical resistance of chemiresistors based on pristine and Pd-modified ZnO NRs to various concentrations (30–1000 ppm) of butane (C_4_H_10_) at an operating temperature of 300 °C.

When pristine and Pd@ZnO-based gas sensors are exposed to butane gas, the sensor response, the change of electrical resistance, of the hybrid sensing layers is about one order of magnitude higher than that of unmodified ZnO NRs. All films show an n-type behavior. Therefore the electrical resistance decreases in the presence of a reducing gas such as C_4_H_10_. The sensor responses increase upon increasing C_4_H_10_ gas concentration, recovering completely to the initial value after the removal of C_4_H_10_ gas in the test cell. As reported in the calibration curves in Figure 4B, the sensing response of pristine and Pd-modified ZnO were strongly influenced by the presence of Pd catalyst on the surface of ZnO NRs.

**Figure 5:**
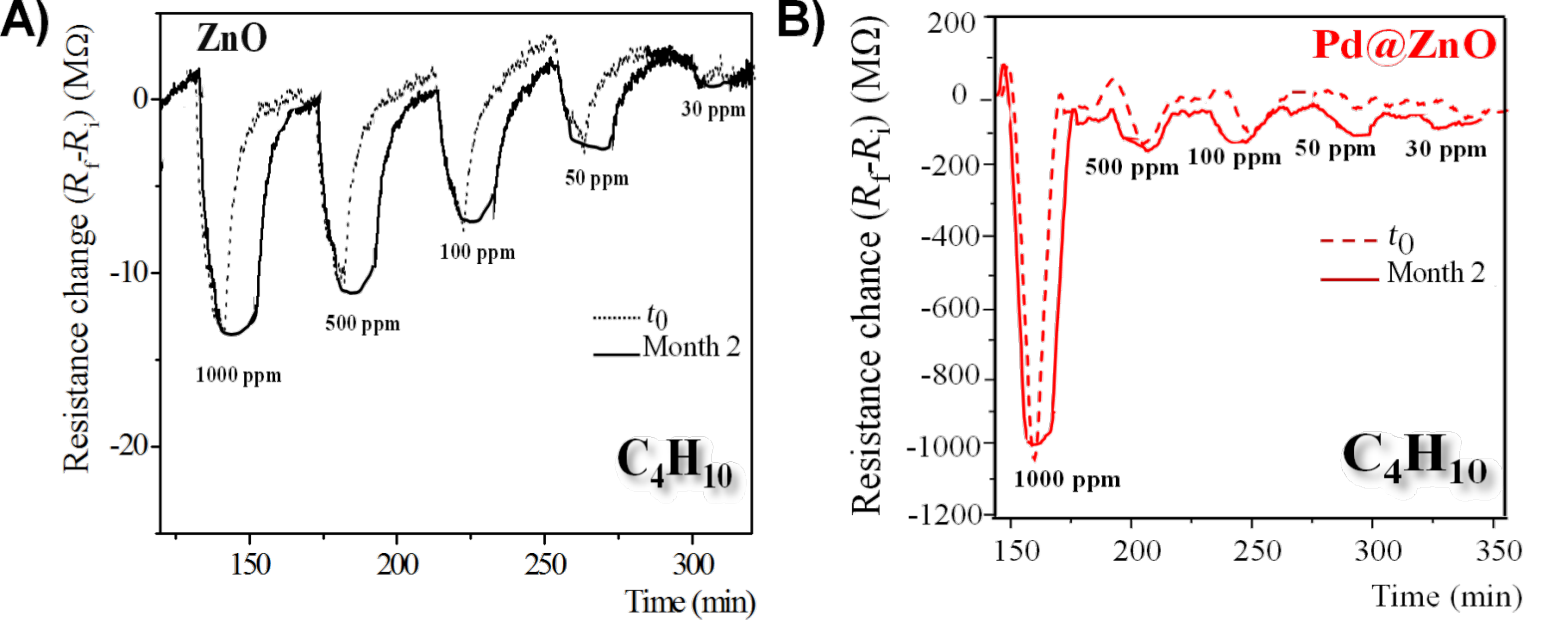
Time response of **A)** pristine ZnO and **B)** Pd-modified ZnO, detected at with as-prepared sensors (*t*_0_) and after two months, exposed to different concentrations of butane (30–1000 ppm) at an operating temperature of 300 °C.

In Figure 5, the sensing responses of pristine and Pd-modified ZnO towards butane at different concentrations (30–1000 ppm), as prepared (*t*_0_) and after a period of two months, were compared. Good reproducibility and stability of the gas sensors over the time are revealed.

In Table 2 the response and recovery times of pristine and Pd-modified ZnO NRs at different butane concentrations are reported.

**Table 2:** Comparison of the response time (t_Response_) and recovery time (t_Recovery_) between pristine and Pd-modified ZnO NRs at various C_4_H_10_ concentrations.

	*t*_response_ (s)		*t*_recovery_ (s)	
*c*(C_4_H_10_) (ppm)	pristine ZnO NRs	Pd-modified ZnO NRs	pristine ZnO NRs	Pd-modified ZnO NRs

1000	445 ± 30	318 ± 30	600 ± 30	355± 30
500	495 ± 30	430 ± 30	641 ± 25	525 ± 30
100	526 ± 30	450 ± 30	645 ± 30	590 ± 30
50	533 ± 30	480 ± 30	700 ± 30	610 ± 30
30	538 ± 30	488 ± 30	730 ± 30	668 ± 30

Over the whole investigated concentration range the response and recovery processes were faster on Pd-modified ZnO NRs. This behavior can be attributed to the presence of Pd NPs, which catalyze the sensing process. Moreover, the response time was faster than the recovery time, in both cases, Pd-modified and pristine ZnO NRs. This is probably because the gas molecules adsorb more quickly on the surface of the sensing layer, while the desorption of gaseous species produced in the sensing process takes longer [48]. The response/recovery times in both cases were longer than those reported in literature for similar gas sensing layers (e.g., Pd-sensitized ZnO nanobeads [48]). This is probably because of the lower film porosity. A high film porosity is necessary to obtain better results with this HCs gas sensing mechanism [49–50].

**Figure 6:**
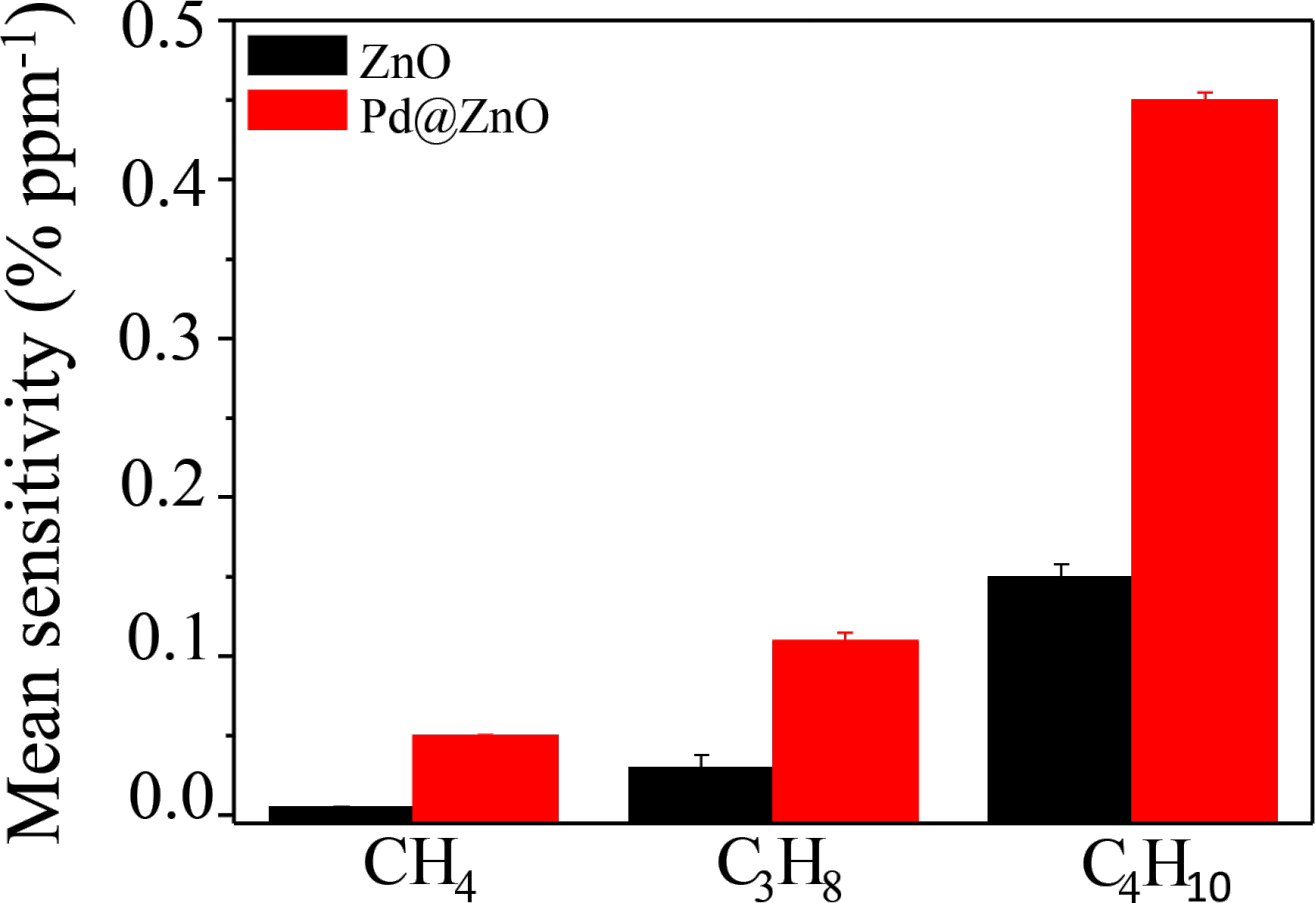
Mean sensitivity of pristine and Pd@ZnO towards CH_4_, C_3_H_8_, and C_4_H_10_ gases at an operating temperature of 300 °C.

To evaluate and compare the cross-sensitivity of the unmodified and Pd-modified ZnO NRs, the mean sensitivity towards methane (CH_4_), propane (C_3_H_8_) and butane (C_4_H_10_) gases at an operating temperature of 300 °C is reported in Figure 6.

The mean sensitivity of Pd-modified ZnO NRs is always higher than that of pristine ZnO for all analyzed HC gases. Pd NPs have a positive catalytic effect on HC gas sensing. Moreover, for both pristine and Pd-modified ZnO NRs, the mean sensitivity increases with the length of the chain length of the hydrocarbon gas. This trend can be explained by the fact that hydrocarbons with longer alkane chains have a higher surface area exposed to the sensing layer [51]. This promotes the gas adsorption process, which is the crucial step in the gas sensing [50].

The enhanced response of the Pd@ZnO NRs can be attributed to the formation of highly reactive species as reported in the following reaction [52]:

[1]
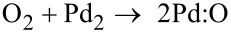


The weak complex formed between Pd atoms and oxygen molecules quickly dissociates producing oxygen atoms that migrate along the surface of ZnO grains. This migration, catalyzed by Pd atoms is well known as spillover of the gas ions. In this way, the oxygen atoms capture electrons from the surface of ZnO and, at the same time, acceptor surface states are formed [40]. The reducing gases react with oxygen on the surface, lowering the electrical resistance of ZnO. In presence of a great number of oxygen species, more reactions take place. The gaseous HC molecules exposed to Pd-modified ZnO NRs react with adsorbed oxygen in the same manner as described in Equation 1. Thus, the sensitivity towards HC gases can be improved by Pd NP catalysts deposited onto ZnO NR surface.

**Figure 7:**
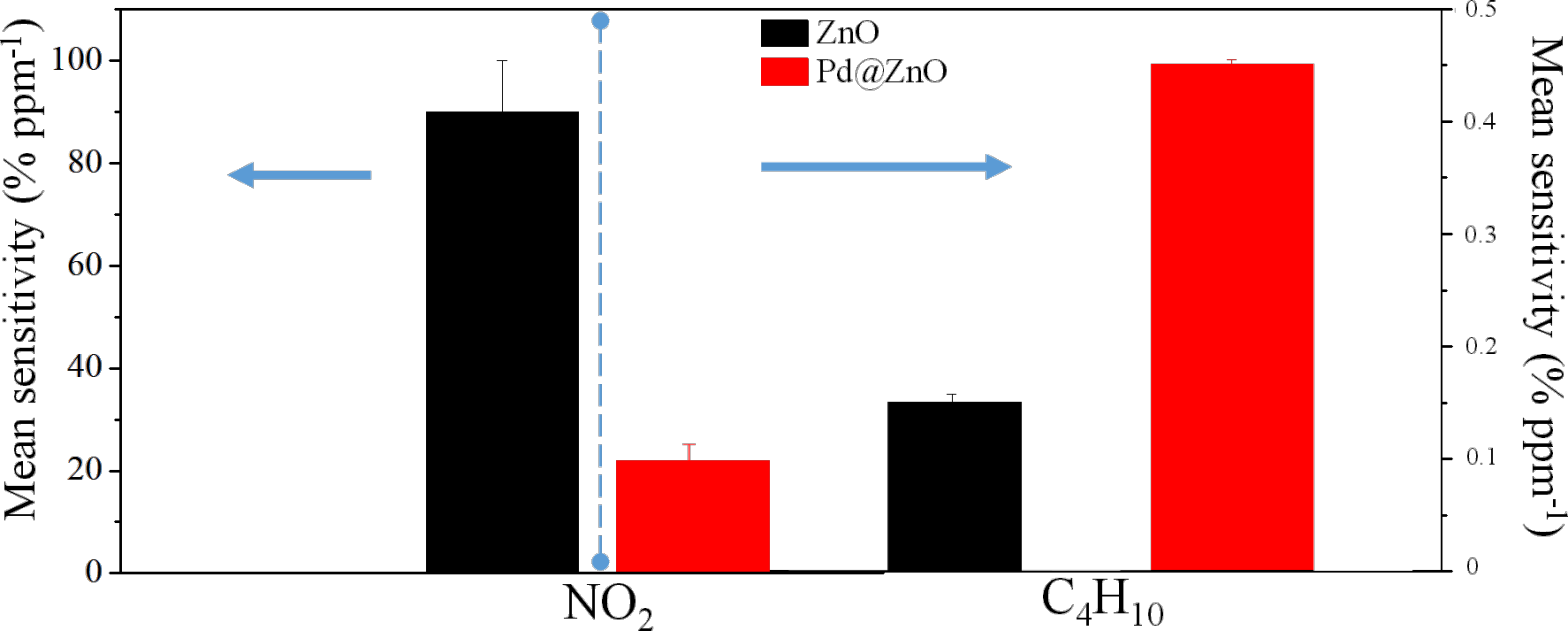
Mean sensitivity of pristine and Pd@ZnO NRs towards NO_2_ and C_4_H_10_ at an operating temperature of 300 °C.

To evaluate the sensor selectivity, the mean sensitivity of pristine and Pd-modified ZnO NRs towards nitrogen dioxide and butane, at an operating temperature of 300 °C, is reported in Figure 7. In NO_2_ gas sensing, the pristine ZnO NRs show a higher response. In contrast, the presence of the Pd NPs on the ZnO NRs improves the selectivity towards C_4_H_10_.

In Figure 7, butane gas has been selected to represent all investigated HCs, since the sensor response towards it is the highest of all investigated HC gases. Then, the high selectivity of Pd@ZnO-based gas sensor towards butane gas can be extended to all HCs gas in presence of NO_2_ gas. The catalytic effect of Pd NPs positively affects the sensing of reducing HCs gases and lowers the detection of oxidizing NO_2_ gas.

When measuring a mixture of HCs gases, since the resistance variation for all investigated HCs is the same, this sensor system does not permit to distinguish the type of HC gas, producing a sum result as sensor response. To overcome this problem a multiplexed array of differently functionalized sensing materials could be used, in which each sensor system is selective in the detection of a specific gas.

## Conclusion

The successful electrochemical functionalization of ZnO NRs by Pd NPs is reported. The gas sensing properties of pristine and Pd-modified rod-like ZnO-based chemiresistor revealed that the presence of catalytic Pd NPs on the surface of ZnO NRs improves the sensitivity and selectivity towards the detection of HCs gases at an operating temperature of 300 °C.

Future work will be addressed to evaluate the sensing properties of electrochemically functionalize ZnO NRs with noble metals (e.g. Au and Pd), used as sensing layer in chemiresistive gas sensors, to improve their sensitivity and selectivity towards other toxic and polluting gases.
